# Shooting Utility Maximization in UAV-Assisted Wireless Camera Sensor Networks

**DOI:** 10.3390/s22103685

**Published:** 2022-05-12

**Authors:** Yulei Wu, Simeng Feng, Chao Dong, Weijun Wang

**Affiliations:** 1 College of Electronic and Information Engineering, Nanjing University of Aeronautics and Astronautics, Nanjing 211106, China; wuyulei@nuaa.edu.cn; 2 College of Computer Science and Technology, Nanjing University, Nanjing 210023, China; weijunwang@smail.nju.edu.cn

**Keywords:** unmanned aerial vehicle (UAV), target shooting utility, trajectory planning, energy consumption

## Abstract

Recently, wireless camera sensor networks (WCSNs) have entered an era of rapid development, and WCSNs assisted by unmanned aerial vehicles (UAVs) are capable of providing enhanced flexibility, robustness and efficiency when executing missions such as shooting targets. Existing research has mainly focused on back-end image processing to improve the quality of captured images, but it has neglected the question of attaining quality images on the front-end, which is significantly influenced by the location and hovering time of the UAV. Therefore, in this paper, we conceive a novel shooting utility model to quantify shooting quality, which is maximized by simultaneously considering the UAV’s trajectory planning, hovering time and shooting point selection. To expound further, we prove the submodularity of the utility function, whereby the original problem can be expressed as a submodular maximization problem with path constraints, and we propose a utility-cost ratio (UCR) algorithm to maximize shooting utility through two-level optimization. Then, by using the relaxation of the cost function, we analyze the gap between the proposed algorithm and the optimal algorithm (OPT) and prove that the UCR algorithm has a bi-criterion approximation ratio of $\left(1-1/e\right)/2$. Simulation results show that the algorithm outperforms both the random algorithm (RAN) and the maximum shooting utility point selection algorithm (MSU) in terms of shooting utility and time utilization efficiency, improving shooting utility by 51% and 21% compared to the RAN and MSU algorithms, respectively, and achieving at least 88.2% of the OPT algorithm in terms of time utilization efficiency.

## 1. Introduction

In recent years, wireless camera sensor networks (WCSNs) have attracted substantial attention from academia and industry, as they can obtain high-resolution target information by providing videos and images [1,2,3]. WCSNs are capable of supporting a wide range of applications, such as traffic monitoring [4,5], building inspection [6] and crowd protection [7], with the aid of camera shooting. However, setting up fixed camera sensors for WCSNs may require extensive infrastructure implementation, resulting in cost inefficiencies.

**Figure 1:**
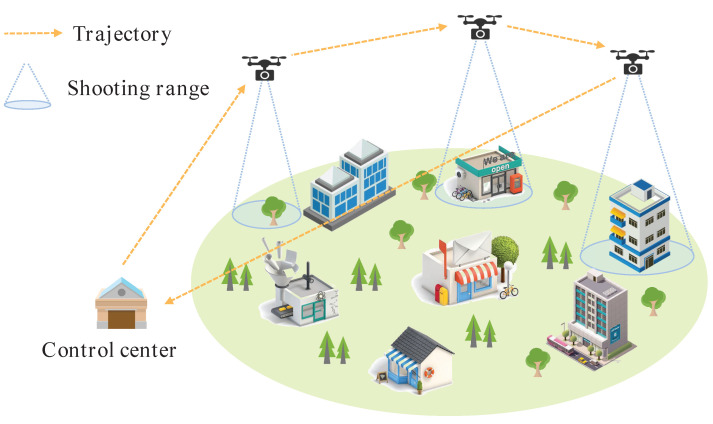
A diagram of a UAV-assisted WCSN scenario.

Fortunately, thanks to the mobility, flexibility and economic feasibility of unmanned aerial vehicles (UAVs), these devices constitute promising candidates for shooting images for WCSNs when the UAVs are equipped with camera sensors [8,9,10,11]. As shown in Figure 1, when shooting and monitoring urban buildings, a traditional fixed camera sensor network cannot cope with challenges such as high altitude or panoramic shooting, whereas UAVs can move toward targets for multi-angle shooting, which greatly improves the efficiency of shooting tasks.

Substantial efforts have been made in the research of UAV-assisted WCSNs, and the existing research mainly focuses on conserving system resources and improving image quality. First, in terms of conserving system resources, some works are mainly devoted to the question of how to reduce the energy and power consumption of UAVs. For example, Gao et al. [12] designed a network of UAV-assisted camera sensors to detect air quality. In order to minimize the energy consumption of the UAVs, they optimized the flight altitude of the UAVs and their two-dimensional coordinates when flying with the aim of ensuring the accuracy of air quality indicators. Ji et al. [13] investigated the problem of using cooperative UAVs in reconnaissance missions to achieve full coverage of targets. They achieved fairness in energy consumption by planning the trajectory of multiple UAVs. Zhan et al. [14] considered the energy consumption problem when using UAVs to collect data from sensor nodes. They jointly optimized the wake-up time of sensor nodes and the trajectory of the UAVs to minimize the maximum energy consumption of all sensor nodes.

In addition, some works mainly focus on improving image quality. Considering the problem of low illumination in UAV pedestrian detection at night, Wang et al. [15] proposed a block-matching and 3D-filtering method to improve the low-illumination image quality. Yuan et al. [16] proposed a seam-cutting strategy based on UAV image mosaics to reduce image distortions and artifacts. Zhang et al. [17] proposed a new real-time pure image mosaic technology for UAVs. They synthesized aerial images through mosaic transformation to obtain wide-angle panoramic images quickly and accurately. Wang et al. [18] considered the scenario of blurred images due to insufficient power when UAVs shoot high-frequency images and proposed a nonlinear image enhancement method to improve the quality of blurred images from UAV aerial photography. However, these studies all focused on back-end image processing, and the influence of the flight trajectory and the hovering time of the UAV on shooting was not considered at the front-end.

As far as we know, most of the existing research on UAV-assisted WCSNs focuses on the back-end processing of UAV-shot images. However, there is little consideration given to the front-end, such as the influence of the shooting time and the hovering position of the UAV on the shot image. With respect to UAV-assisted data acquisition, we know that the hovering position and hovering time of UAVs have a significant influence on the information collected [19,20]. Based on this principle, in this paper, we conceive the concept of a shooting utility to quantify the amount of information obtained. Then, the UAV’s trajectory planning, hovering time and shooting point selection are optimized to maximize shooting utility. The main contributions of this work are as follows:

- We conceive a novel shooting utility model to quantify shooting quality and then address the problem of maximizing shooting utility. We show that the elaborated shooting utility function obeys the rule of submodularity, which is beneficial for transforming the original problem into a submodularity maximization problem with path constraints.
- We formulate a utility-cost ratio (UCR) algorithm to maximize shooting utility. By using the relaxation of the cost function, we analyze the gap between the proposed algorithm and the optimal algorithm (OPT) and prove that the UCR algorithm has a bi-criterion approximation ratio of $\left(1-1/e\right)/2$.
- We conduct numerous simulations to analyze the performance of the UCR algorithm. The results demonstrate that the algorithm outperforms both the random algorithm (RAN) and the maximum shooting utility point selection algorithm (MSU) in terms of shooting utility and time utilization efficiency, improving shooting utility by 51% and 21% compared to the RAN and MSU algorithms, respectively, and achieving at least 88.2% of the OPT algorithm in terms of time utilization efficiency.

The rest of this paper is organized as follows. In Section 2, we describe the model of the UAV-enabled shooting system in detail. In Section 3, we analyze the problem and put forward a solution. Section 4 provides the simulation results and performance evaluation. Finally, we summarize this paper in Section 5.

## 2. System Model

**Figure 2:**
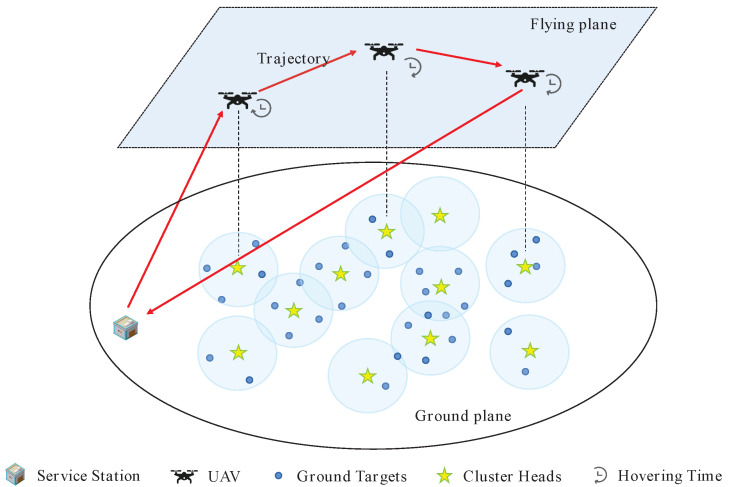
A diagram of the shooting scenario.

We consider a UAV-enabled shooting mission scenario, as shown in Figure 2. Assume that we have a total of *M* targets $O=\{o_{1},o_{2},\ldots ,o_{m}\}$ on the ground that must be shot. A UAV equipped with a camera is capable of shooting an area with radius *R*. Since the ground targets are widely distributed, it is expensive, time-consuming and difficult for the UAV to fly over each target for its shooting mission. Therefore, we adopt an overlapping clustering approach [21,22] to group the ground targets into different clusters to accomplish the shooting mission efficiently, and the cluster head set is denoted as $C=\{c_{1},c_{2},\ldots ,c_{n}\}$. By doing so, the UAV is capable of moving to the position above the cluster head to complete the shooting of members in the cluster, which means that the UAV can capture multiple targets at a single shooting point, and a target can be captured at multiple shooting points. We study the scenario of a UAV flying at altitude *h* with a fixed speed $v_{a}$. The UAV starts at the service station $s_{0}$ and returns to $s_{0}$ after completing the shooting tasks. The major notations used in this paper are summarized in Table 1.

### 2.1. Energy Consumption Model

We divide the energy consumption model into two categories: one is motion energy cost, and the other is hovering energy cost. The motion energy is related to the flight distance of the UAV between clusters, while the hovering energy is related to the hovering time of the UAV at each cluster head. We can use $(x\left(c_{i}\right),\left(y\left(c_{i}\right)\right)$ to denote the location coordinates of the cluster head $c_{i}$; then, the Euclidean distance between two cluster heads can be expressed as

(1) $$d\left(c_{i},c_{i+1}\right)=\sqrt{{\left[x\left(c_{i+1}\right)-x\left(c_{i}\right)\right]}^{2}+{\left[y\left(c_{i+1}\right)-y\left(c_{i}\right)\right]}^{2}}.$$

We use the set K to represent the cluster heads over which the UAV chooses to hover; then, a new selected cluster head set K⊆C can be formed and used to obtain the following motion energy cost

(2) $$E_{m}=\sum_{d\left(k_{i^{\prime }},k_{i^{\prime }+1}\right)\in L^{\mathrm{TSP}}\left(K\right)}\frac{p_{m}d\left(k_{i^{\prime }},k_{i^{\prime }+1}\right)}{\eta v_{a}},$$

where $d(k_{i^{\prime }},k_{i^{\prime }+1})$ represents the distance between two adjacent points in the set K, $L^{\mathrm{TSP}}\left(K\right)$ is a closed path for the UAV that starts at and returns to the service station $s_{0}$, $p_{m}$ is the minimum motion power consumption of the UAV and η is the UAV’s power efficiency. We use the parameter λ to represent $p_{m}/\eta v_{a}$. The UAV only visits each cluster head in K once.

The UAV’s hovering energy consumption is related to the hovering time and various parameters of the UAV. We use $t_{i}$ to denote the time that UAV hovers at the cluster head $c_{i}$. Then, we denote the hovering energy consumption for cluster head $c_{i}$ as

(3) $$E\left(c_{i}\right)=\sqrt{\frac{{\left(mg\right)}^{3}}{2\pi \rho r_{u}^{2}n_{u}}}t_{i},$$

where *m*, *g* and ρ are, respectively, the mass of the UAV, the gravity of the earth and the air density. The radius of the rotor and the number of rotors are expressed by $r_{u}$ and $n_{u}$, respectively. We use parameter μ to represent $\sqrt{{\left(mg\right)}^{3}/2\pi \rho r_{u}^{2}n_{u}}$. For the selected cluster head set K, the total hovering energy consumption can be expressed as

(4) $$E_{h}=\sum_{c_{i}\in K}E\left(c_{i}\right)=\sum_{c_{i}\in C}\sqrt{\frac{{\left(mg\right)}^{3}}{2\pi \rho r_{u}^{2}n_{u}}}t_{i}a_{i},$$

where $a_{i}$ is a binary variable used to indicate whether the UAV chooses to hover above the cluster head $c_{i}$ for shooting. For example, $a_{i}$ is 1 if $c_{i}$ is selected; otherwise, it is 0. Thus, the total cost for any selected cluster head set K can is given by

(5) $$\begin{array}{cc} E(K) & =E_{m}+E_{h}\\ \; & =\sum_{d\in L^{TSP}\left(K\right)}\frac{p_{m}d}{\eta v_{a}}+\sum_{c_{i}\in C}\sqrt{\frac{{\left(mg\right)}^{3}}{2\pi \rho r_{u}^{2}n_{u}}}t_{i}a_{i}\\ \; & =\sum_{d\in L^{TSP}\left(K\right)}\lambda d+\sum_{c_{i}\in C}\mu t_{i}a_{i}. \end{array}$$

### 2.2. Shooting Utility Model

As the shooting time is related to acquired information, we formulate the shooting utility model to quantify the amount of information we obtain. For the shooting utility model, we first define the shooting utility weight $p_{ij}$, that is, the amount of information that can be obtained per unit time by shooting a cluster member $o_{j}$ above the cluster head $c_{i}$. Then we find that the shooting utility of the UAV flying above the cluster head $c_{i}$ on target $o_{j}$ is

(6) $$q\left(o_{j},c_{i}\right)=p_{ij}t_{ij},$$

where $t_{ij}$ is the shooting time allocated to the target $o_{j}$ when the UAV hovers over the cluster head $c_{i}$. According to overlapping clustering, a target may belong to multiple clusters, and the utility of a target contributed by multiple clusters is additive. That is to say, for any given set K⊆C, the total shooting utility for target $o_{j}$ can be expressed as

(7) $$q_{K}\left(o_{j}\right)=\sum_{c_{i}\in K}q\left(o_{j},c_{i}\right)=\sum_{c_{i}\in C}p_{ij}t_{ij}a_{i}.$$

Furthermore, considering the varying importance of each target, the maximum amount of information that can be captured also varies, so that there is a maximum shooting utility $Q_{j}^{m}$ defined for each target. When the shooting time is greater than a predefined threshold, shooting utility remains equal to $Q_{j}^{m}$. Thus, we can obtain the complete shooting utility of the target $o_{j}$ as

(8) $$Q\left(K,o_{j}\right)=\min\left\{q_{K}\left(o_{j}\right),Q_{j}^{m}\right\}.$$

In order to simplify the model, in this paper, we do not consider the correlation between targets or the information redundancy when shooting the same target from different positions. We think that shooting utility is a simple addition quantity, but in this work, we consider it according to the feature distribution of computer vision and use information theory modeling to solve this problem [23].

## 3. Problem Formulation

The total shooting utility is the sum of the shooting utility of UAVs for all members in the selected clusters, which can be expressed as

(9) $$Q\left(K,T\right)=\sum_{j=1}^{m}Q\left(K,o_{j}\right)=\sum_{j=1}^{m}\min\left\{q_{K}\left(o_{j}\right),Q_{j}^{m}\right\}=\sum_{j=1}^{m}\min\left\{\sum_{c_{i}\in C}p_{ij}t_{ij}a_{i},Q_{j}^{m}\right\},$$

in which $T=\left\{t_{ij}|i=1,\cdots ,n;j=1,\cdots ,m\right\}$ represents the shooting time allocation of the UAV to targets in different clusters. As the sum of shooting time assigned to cluster members cannot be greater than the hover time of the UAV at the cluster head position, we have

(10) $$\sum_{o_{j}\in O}t_{ij}a_{i}\leq t_{i},\forall c_{i}\in C.$$

At the same time, considering the energy constraints of the UAV, the total energy consumption cannot exceed its maximum energy consumption, which can be described as

(11) $$E\left(K\right)\leq E_{\max},\forall K\subseteq C,$$

where $E_{\max}$ is the maximum energy consumption of the UAV.

As defined above, our goal is to find a closed path for the UAV, that is, to find a cluster head subset K that maximizes the shooting utility of the UAV on a target in the network under the conditions expressed in Equations (10) and (11). Thus we can describe our problem as

(12) $$\begin{array}{cc} P1:\; & \max_{a_{i},t_{ij}}Q\left(K,T\right)\\ s.t.\; & \sum_{o_{j}\in O}t_{ij}a_{i}\leq t_{i},\forall c_{i}\in C,\\ \; & E\left(K\right)\leq E_{\max},\forall K\subseteq C. \end{array}$$

The cluster head set K and time allocation strategy T can be found by optimizing $a_{i}$ and $t_{ij}$.

According to the above definition, the formulated problem consists of a two-layer optimization, where the optimization objective at the bottom layer is cluster head selection and time allocation, and the top layer performs trajectory planning for the energy-constrained UAV based on the selected cluster heads. The two layers of optimization are interrelated and cannot be handled separately to obtain the global solution. If the cluster head is selected without considering trajectory planning, a lot of motion energy is consumed. If the appropriate cluster head is not considered in trajectory planning, the shooting utility cannot be maximized.

When considering only shooting utility without considering the UAV energy constraint, we can reduce the problem P1 to a 0-1 backpack problem, as follows:

(13) $$\begin{array}{cc} \; & \max_{a_{i},t_{ij}}Q\left(K,T\right)\\ s.t.\; & \sum_{o_{j}\in O}t_{ij}a_{i}\leq t_{i},\forall c_{i}\in C, \end{array}$$

The 0-1 backpack problem is a classic NP-hard problem. When we further consider the energy consumption of the UAV, the problem can be modeled as a traveling salesman problem (TSP). It is intractable to attain the traveling cost E(K), as it needs to find a closed TSP tour $L^{\mathrm{TSP}}\left(K\right)$, which is also an NP-hard problem. Therefore, the formulated optimization problem P1 is coupled by two NP-hard problems.

## 4. An Effective Approximation Algorithm: UCR Algorithm

In this section, we first prove the submodularity of the utility function, based on which we can formulate the problem as a submodular maximization problem with path constraints and propose an efficient approximation algorithm, that is, a UCR, to maximize the shooting utility of the UAV.

### 4.1. A Proposed UCR Algorithm

In order to solve our problem, we first prove that our function has three properties: monotonicity, nonnegativity and submodularity. These properties are beneficial because they allow us to transform our problem into a submodule maximization problem with path constraints. For convenience of expression, we construct a function G(K) to represent our shooting utility function. We obtain the equational relation $G\left(K\right)=Q\left(K,T^{\prime }\right)$, which means that when the time allocation strategy is $T^{\prime }$, the total shooting utility is G(K). Then we prove that the function G(K) has the above three properties.

**Definition** **1.** *For a given element set Φ, a real set function is defined as f:$2^{\Phi }\to R$. f is considered to be monotonous, nonnegative and submodular if and only if f satisfies the following conditions, respectively:*

- *f(A)≤f(B) for all A⊆B⊆Φ or equivalently: $f\left(A\cup \left\{x\right\}\right)-f\left(A\right)>0$ for all A⊆Φ and x∈Φ\A (monotonicity);*
- *f(⌀)=0 and f(A)≥0 for all A⊆Φ (nonnegativity);*
- *f(A)+f(B)≥f(A∪B)+f(A∩B) for any A,B⊆Φ or equivalently: $f\left(A\cup \left\{x\right\}\right)-f\left(A\right)\geq f\left(B\cup \left\{x\right\}\right)-f\left(B\right)$ for all A⊆B⊆Φ,x∈Φ\B (submodularity).*

**Theorem** **1.** *The constructed function G(K) is monotone, nonnegative and submodular.*

**Proof**  The detailed procedure is shown in Appendix A.    □

On the basis of proving that G(K) is a submodule function, we design an effective algorithm that considers two-level optimization to solve the submodule maximization problem with path constraints by referring to the idea in [24,25].

For the cluster head $c_{i}$ in C, we sort the ground targets in order of decreasing utility weights. The UAV then greedily allocates the shooting time to each target in this order until each target rises to the utility threshold or the UAV has no remaining time to allocate at the cluster head $c_{i}$ position. That is to say, before shooting utility reaches the threshold, we tend to allocate more time to shoot targets with higher utility weights. Furthermore, we need to take into account the energy constraints of the UAV. In each iteration, we use the nearest neighbor rule to calculate the energy consumption of the UAV. The core of this algorithm is that for a given set K, we need to add a new cluster head $c_{i^{\prime }}$ with the maximum utility-cost ratio to the current set. The newly added cluster head can be represented as

(14) $$c_{i^{\prime }}=\underset{c\in C\backslash K_{i-1}}{\arg\max}\frac{G\left(K_{i-1}+c\right)-G\left(K_{i-1}\right)}{\hat{E}\left(K_{i-1}+c\right)-\hat{E}\left(K_{i-1}\right)},$$

where $\hat{E}\left(\cdot \right)$ is an approximate cost function of E(·). The reason for this substitution is that we observe that the optimal cost function cannot be calculated in polynomial time, so we use an effective approximate cost function in its place. This may influence the quality of our results. Therefore, in the next section, we prove the approximation ratio of the algorithm.

The detailed steps of the UCR algorithm can be found in Algorithm 1. In each iteration, we use the greedy method to select a cluster head with the largest utility-cost ratio. Using this iterative method, we can continuously add new cluster heads to the set K to obtain higher shooting utility until the energy consumption constraint of the UAV is broken.

### 4.2. Approximation Analysis

One of the major limitations of prior works on submodular maximization with routing constraints is that the cost function was usually assumed to be a submodule. However, in many practical problems, the optimal cost function is not submodular. To solve this problem, we take advantage of the natural relaxation of the cost function, the β-submodularity [26], and the approximation of our algorithm is affected by the β-submodularity.

**Definition** **2.** *For the energy consumption cost function E, β-submodularity is defined as*

(15) $$\beta =\min_{c}\min_{A,B:A\subset B}\frac{E(A+c)-E(A)}{E(B+c)-E(B)}.$$

**Algorithm 1:** Utility-Cost Ratio Algorithm
**Input:** 
Cluster head set $C=\{c_{1},c_{2},\ldots ,c_{n}\}$, target set $O=\{o_{1},o_{2},\ldots ,o_{m}\}$, energy constraint ${\hat{E}}_{\max}$, shooting utility weight $p_{ij}$.**Output:** 
Selected cluster heads K⊆C, time allocation T.  1:Initialize $C^{\prime }\leftarrow C$, T←⌀, $K_{0}\leftarrow ⌀$, $\hat{E}\left(K_{0}\right)\leftarrow 0$, i←1, $K^{\prime }=\arg\max\left\{G\left(c\right)|c\in C,\hat{E}\left(c\right)\leq E_{\max}\right\}$;  2:**while** $C^{\prime }\neq ⌀$ **do**  3:    **for** $c\in C^{\prime }$ **do**  4:        Calculate $G(K_{i-1}+c)$ and $G(K_{i-1})$ with corresponding time allocation scheme;  5:        Use the nearest neighbour rule to calculate the energy consumption $\hat{E}\left(K_{i-1}+c\right)$ and $\hat{E}\left(K_{i-1}\right)$;  6:    **end for**  7:    $c_{i^{\prime }}=\underset{c\in C\backslash K_{i-1}}{\arg\max}\frac{G\left(K_{i-1}+c\right)-G\left(K_{i-1}\right)}{\hat{E}\left(K_{i-1}+c\right)-\hat{E}\left(K_{i-1}\right)}$;  8:    **if** $\hat{E}\left(K_{i-1}+c_{i^{\prime }}\right)\leq E_{\max}$ **then**  9:        $K_{i}\leftarrow K_{i-1}+c_{i^{\prime }}$, i←i+1, $T\leftarrow T_{c_{i^{\prime }}}$;10:    **end if**11:    $C^{\prime }\leftarrow C^{\prime }\backslash c_{i^{\prime }}$;12:
**end while**
13:**if** $G\left(K^{\prime }\right)\geq G\left(K_{i-1}\right)$ **then**14:     $K\leftarrow K^{\prime }$;15:
**else**
16:      $K\leftarrow K_{i-1}$;17:
**end if**
18:Output K⊆C, T


In addition, for the cost function *E*, we need another concept curvature to help prove the approximate ratio, which essentially measures the linear deviation.

**Definition** **3.** *For the cost function E in the subsets of *Ψ*, we can define the total curvature ${\bar{K}}_{e}$ as*

(16) $${\bar{K}}_{e}=1-\min_{c\in \Psi }\frac{E(\Psi \backslash c+c)-E(\Psi )}{E(c)}.$$

**Definition** **4.** *We introduce another variable $N_{e}$, which is the largest set of feasible solutions for our problem in the set *Ψ*, defined as*

(17) $$N_{e}=\max\left\{|K|:E\left(K\right)\leq E_{\max}\right\}.$$

In the UCR algorithm, the initial set is $K_{0}=0$; through iteration, we can continuously add new cluster heads to the set K, thus generating a series of intermediate sets $K_{1},...,K_{s}$ until the energy consumption constraint of UAV is broken. We assume that the s+1 iteration does not satisfy the constraint, and then the iteration stops.

**Lemma** **1.** *For i=1,...,s+1, it holds that*

(18) $$G\left(K_{i}\right)\geq \left[1-\prod_{l=1}^{i}\left(1-\frac{\hat{E}\left(K_{l}\right)-\hat{E}\left(K_{l-1}\right)}{E_{\max}}\right)\right]G\left(\tilde{K}\right).$$

**Proof.**  For i=1,...,s+1,

(19) $$G\left(K_{i}\right)-G\left(K_{i-1}\right)\geq \frac{\hat{E}\left(K_{i}\right)-\hat{E}\left(K_{i-1}\right)}{E_{\max}}G\left(\tilde{K}\right)-G\left(K_{i-1}\right)),$$

where $\hat{E}$ is a β submodular ψ(n)-approximation of the β-submodular cost function *E*, and $\tilde{K}$ is the optimal solution of $\max\left\{G\left(K\right)|E\left(K\right)\leq \beta \frac{E_{\max}\left(1+\beta \left(N_{e}-1\right)\left(1-{\bar{K}}_{e}\right)\right)}{\psi \left(n\right)N_{e}}\right\}$ [26].

Thus for i=1, we can obtain

(20) $$G\left(K_{1}\right)\geq \frac{\hat{E}\left(K_{1}\right)-\hat{E}\left(K_{0}\right)}{E_{\max}}G\left(\tilde{K}\right).$$

For i>1, we can obtain

(21) $$\begin{array}{cc} G(K_{i}) & =G\left(K_{i-1}\right)+\left[G\left(K_{i}\right)-G\left(K_{i-1}\right)\right]\\ \; & \geq G\left(K_{i-1}\right)+\frac{\hat{E}\left(K_{i}\right)-\hat{E}\left(K_{i-1}\right)}{E_{\max}}\left(G\left(\tilde{K}\right)-G\left(K_{i-1}\right)\right)\\ \; & =\left(1-\frac{\hat{E}\left(K_{i}\right)-\hat{E}\left(K_{i-1}\right)}{E_{\max}}\right)G\left(K_{i-1}\right)+\frac{\hat{E}\left(K_{i}\right)-\hat{E}\left(K_{i-1}\right)}{E_{\max}}G\left(\tilde{K}\right)\\ \; & \geq \left(1-\frac{\hat{E}\left(K_{i}\right)-\hat{E}\left(K_{i-1}\right)}{E_{\max}}\right)\left[\left(1-\prod_{l=1}^{i}\left(1-\frac{\hat{E}\left(K_{l}\right)-\hat{E}\left(K_{l-1}\right)}{BE_{\max}}\right)\right)G\left(\tilde{K}\right)\right]\\ \; & +\frac{\hat{E}\left(K_{i}\right)-\hat{E}\left(K_{i-1}\right)}{E_{\max}}G\left(\tilde{K}\right)\\ \; & =\left(1-\prod_{l=1}^{i}\left(1-\frac{\hat{E}\left(K_{l}\right)-\hat{E}\left(K_{l-1}\right)}{E_{\max}}\right)\right)G\left(\tilde{K}\right). \end{array}$$

□

**Theorem** **2.** *The UCR algorithm can achieve a bi-criterion approximation ratio of $\left(1-1/e\right)/2$.*

**Proof.**  From Lemma 2, we can obtain

(22) $$G\left(K_{s+1}\right)\geq \left[1-\prod_{l=1}^{s+1}\left(1-\frac{\hat{E}\left(K_{l}\right)-\hat{E}\left(K_{l-1}\right)}{E_{\max}}\right)\right]G\left(\tilde{K}\right)$$

From the fact that the s+1 iteration violates the UAV energy consumption constraint, we have $\hat{E}\left(K_{s+1}\right)>E_{\max}$. Then we can obtain

(23) $$G\left(K_{s+1}\right)\geq \left[1-\prod_{l=1}^{s+1}\left(1-\frac{\hat{E}\left(K_{l}\right)-\hat{E}\left(K_{l-1}\right)}{\hat{E}\left(K_{s+1}\right)}\right)\right]G\left(\tilde{K}\right)$$

For $z_{1},...,z_{n}\in R^{+}$ such that $\sum z_{i}=Z$, $1-\prod_{i=1}^{n}\left(1-z_{i}/Z\right)$ achieves minimum value at $z_{1}=...=z_{n}=Z/n$. Therefore, we have

(24) $$\begin{array}{cc} G(K_{s+1}) & \geq \left[1-\prod_{l=1}^{s+1}\left(1-\frac{\hat{E}\left(K_{l}\right)-\hat{E}\left(K_{l-1}\right)}{\hat{E}\left(K_{s+1}\right)}\right)\right]G\left(\tilde{K}\right)\\ \; & \geq \left[1-{\left(1-\frac{1}{s+1}\right)}^{s+1}\right]G\left(\tilde{K}\right)\geq \left(1-\frac{1}{e}\right)G\left(\tilde{K}\right) \end{array}$$

□

In addition, since we proved that G(K) is a submodular function in the above, we have $G\left(K_{s+1}\right)-G\left(K_{s}\right)\leq G\left(K_{s+1}\right)\leq G\left(K^{*}\right)$, where $K^{*}={\arg\max}_{K\in \Psi }G\left(K\right)$. Therefore, we can obtain

(25) $$G\left(K_{s}\right)+G\left(K^{*}\right)\geq G\left(K_{s+1}\right)\geq \left(1-\frac{1}{e}\right)G\left(\tilde{K}\right)$$

and

(26) $$\max\left\{G\left(K_{s}\right),G\left(K^{*}\right)\right\}\geq \frac{1}{2}\left(1-\frac{1}{e}\right)G\left(\tilde{K}\right).$$

To sum up, the UCR algorithm has a bi-criterion approximation ratio of $\left(1-1/e\right)/2$.

## 5. Simulations and Evaluations

In this section, we simulate the system under different settings and evaluate our algorithm in terms of total shooting utility and time utilization efficiency. The overall shooting utility was introduced in the system model, and the time utilization efficiency can be obtained by dividing the total shooting time of the UAV for the target at the shooting point by the total hovering time, which can be expressed by the formula $\sum_{j=1}^{m}\sum_{i=1}^{n}t_{ij}/\sum_{i=1}^{n}t_{i}$. By default, the task area is 500 m × 500 m in size, the numbers of ground targets and cluster heads are 40 and 10, respectively, and the maximum shooting utility per target is 220. Some other important parameters of the simulation are shown in Table 2 [27,28]. In each different parameter setting, we conducted 1000 simulations and took the average value to obtain the following experimental results.

In order to evaluate the performance of our proposed UCR algorithm, we compare it with the following three algorithms:

- Random algorithm (RAN): Within the energy consumption constraint, the UAV randomly selects cluster heads for shooting.
- Maximum shooting utility point selection algorithm (MSU): The essence of the MSU algorithm is to select the cluster head with the greatest shooting utility in each iteration. This process is repeated until the UAV’s energy consumption exceeds the UAV’s energy capacity or all cluster heads have been selected.
- Optimal algorithm (OPT): We use the powerful optimization software LINGO to obtain the optimal solution to analyze the gap between our proposed algorithm and the optimal one. Because of the high computational complexity, we run it once for different settings to obtain the optimal solution, and we can only use it to solve small instances of this problem.

### 5.1. Impact of the Number of Targets

**Figure 3:**
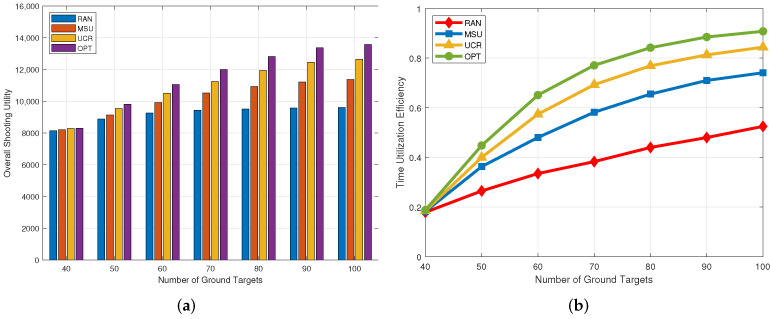
Impact of the number of targets. (**a**) Overall shooting utility; (**b**) Time utilization efficiency.

In Figure 3, the number of ground targets varies from 40 to 100, and we analyze the effects of different numbers of ground targets on shooting utility and time utilization efficiency. It can be seen from Figure 3a that when the number of ground targets is 40, the performance of the UCR algorithm is almost close to the optimal scheme. With an increase in the number of ground targets, the gap between the performance of the UCR algorithm and the OPT algorithm increases from 7.0% to 16.3%. Nevertheless, our proposed algorithm still outperforms the RAN and MSU algorithms in the case of a large number of ground targets. Compared to the RAN algorithm, our algorithm improves shooting utility by 25–50.1%, which is reasonable because RAN randomly selects cluster heads while our algorithm selects cluster heads with the maximum utility-cost ratio at each iteration, taking into account the energy consumption of the UAV, thus saving motion energy and improving shooting utility. Compared to the MSU algorithm, the performance of this algorithm is improved by 14.9–21.9%. This is reasonable because the MSU algorithm only selects the cluster head with the greatest shooting utility in each iteration, while ignoring the energy consumption of the UAV, which leads to higher energy consumption. As shown in Figure 3b, the utilization rate of the hover time of the UAV gradually increases with an increase in ground targets, which is reasonable because when there is a sufficient number of ground targets, shooting utility no longer increases significantly. When the number of ground targets is 100, the optimal scheme can achieve a time utilization rate of 90.8%, and our algorithm can achieve a time utilization rate of 84.4%, which is better than 74.1% for MSU and 52.5% for RAN.

### 5.2. Impact of the Radius of Clusters

**Figure 4:**
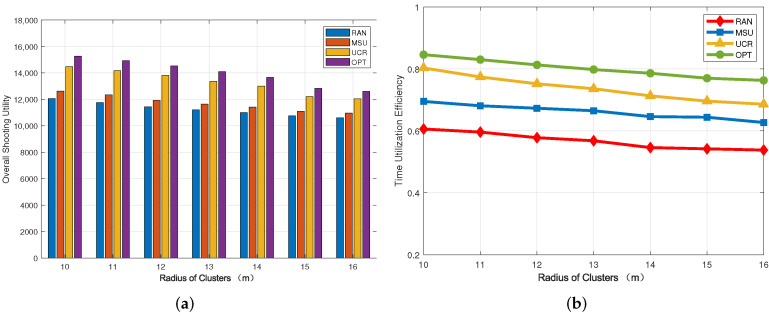
Impact of the number of cluster heads. (**a**) Overall shooting utility; (**b**) Time utilization efficiency.

Figure 4 shows the trend of shooting utility and time utilization efficiency with the radius of clusters. We set the number of ground targets to 40. It can be seen from Figure 4a that with an increase in the radius of the clusters, shooting utility gradually decreases. We note that the average shooting utility of our algorithm is 28.5% higher than that of RAN and 24% higher than that of MSU. In order to compare the gap between our algorithm and the OPT algorithm more intuitively, we give approximations of the shooting utility of the two algorithms for different cluster radius parameters in Table 3. In Figure 4b, we can see that the time utilization shows little change with an increase in the radius of clusters and shows a downward trend as a whole. At the same time, we can still see that the UCR algorithm is the closest to OPT. When the radius of clusters is 10 m, our algorithm achieves a time utilization rate of 80.3%, which is higher than 69.5% for MSU and 60.6% for RAN.

### 5.3. Impact of the UAV Energy Capacity

**Figure 5:**
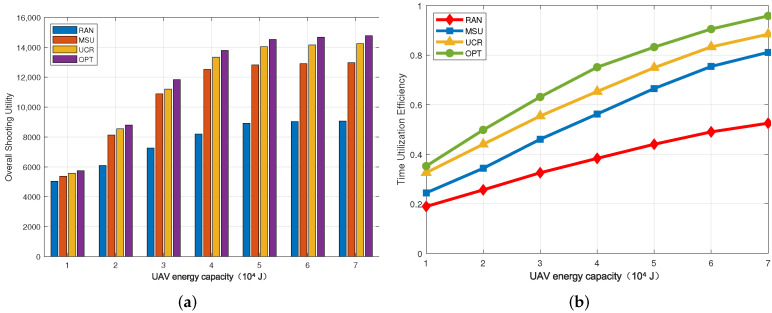
Impact of the UAV energy capacity. (**a**) Overall shooting utility; (**b**) Time utilization efficiency.

In Figure 5, the UAV energy capacity changes from $10^{4}$ J to $7\times 10^{4}$ J. Figure 5a shows that when the energy capacity of the UAV increases, the target shooting utility of the four algorithms is improved. Moreover, the overall shooting utility UCR outperforms MSU and RAN on average by 11.2% and 57.3%, respectively. This is reasonable because, with the growth of the UAV’s energy capacity, more cluster heads can be selected for shooting. As shown in Figure 5b, with the increase in the maximum energy of the UAV, the time utilization efficiency shows an upward trend. It can be seen that when the energy capacity of the UAV is $5\times 10^{4}$ J, the UCR algorithm can achieve a time utilization rate of 74.9%, which is close to the 83.2% of the OPT algorithm and better than 66.5% for MSU and 44% for RAN.

### 5.4. Impact of the Target Distribution Density

**Figure 6:**
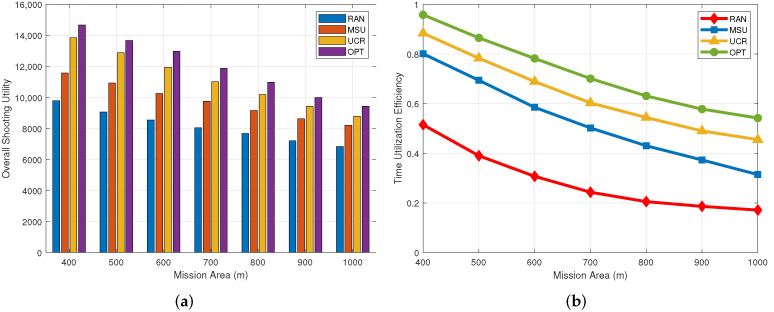
Impact of the target distribution density. (**a**) Overall shooting utility; (**b**) Time utilization efficiency.

Figure 6 shows the trend of target shooting utility and time utilization efficiency with the target distribution density. Assuming that the mission area is square, we set the number of ground targets to 60 and change the side length of the area from 400 m to 1000 m. In Figure 6a, we can see that as the side length of the mission area increases, shooting utility shows a downward trend. This is reasonable because when the number of targets is fixed, a larger mission area means a greater distance between target points and, therefore, a more scattered ground target distribution, which results in more energy consumed for UAV movement and less energy for shooting. As shown in Figure 6a, the UCR algorithm shows better performance with respect to shooting utility. For example, when the side length of the mission area is 400 m, the shooting utility of this algorithm is improved by 20% and 41.1%, respectively, compared with the MSU and RAN algorithms. As shown in Figure 6b, the time utilization shows a decreasing trend as the task area expands. This is reasonable because when the mission area increases, the distribution of ground targets is scattered, and there are fewer targets in each cluster, so the shooting time of the UAV when hovering at the cluster head decreases. When the side length of the mission area is 400 m, our algorithm achieves a time utilization rate of 88.4%, which is higher than 80.1% for MSU and 51.5% for RAN.

### 5.5. Impact of the UAV Parameters

**Figure 7:**
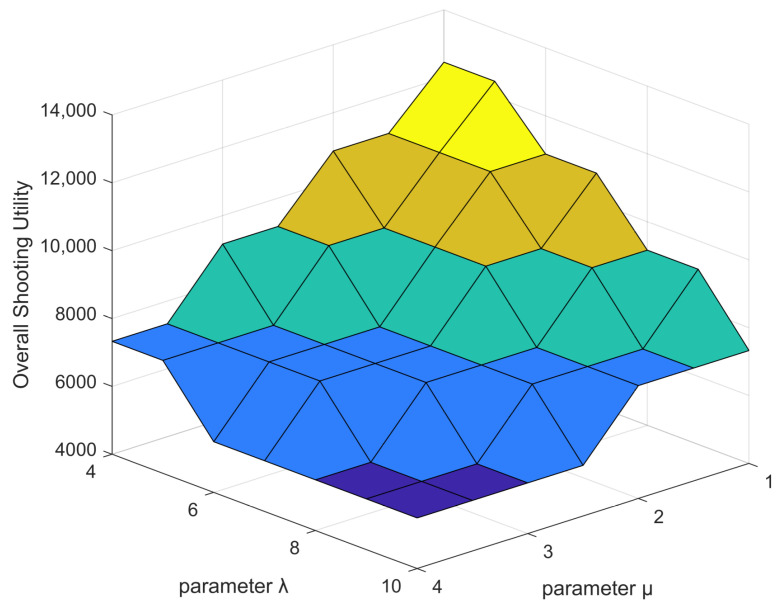
Overall shooting utility versus parameters λ and μ.

The parameters of the UAV have an impact on its motion energy consumption and hovering energy consumption. For example, differences in the weight, flight speed and number of rotors of the UAV yield different results. As shown in Figure 7, we set λ from 4 to 10 and μ from 1 to 4 to evaluate the effect on the overall shooting utility. We can see that smaller values for λ and μ result in higher shooting utility, which is reasonable because λ and μ represent the energy consumption per unit distance of UAV flight and the energy consumption per unit time of UAV hovering, respectively. Therefore, lower values for these variables result in a reduction in energy consumption and an increase in shooting utility.

### 5.6. Trajectory Planning of the UAV

**Figure 8:**
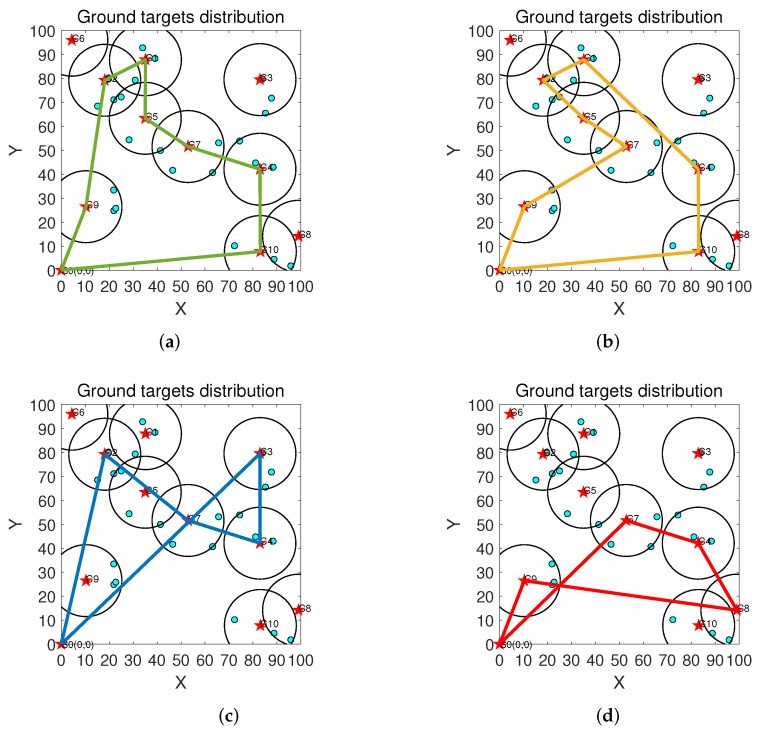
Trajectory planning of the UAV. (**a**) OPT algorithm; (**b**) UCR algorithm; (**c**) MSU algorithm; (**d**) RAN algorithm.

In this section, we use Figure 8 to represent the UAV trajectory generated by each algorithm. From the figure, we see that our algorithm considers the energy consumption of the UAV in each iteration, unlike the MSU and RAN algorithms, so more cluster heads are selected, and the trajectory obtained by our algorithm does not produce a loop. In addition, we can also see that the cluster heads chosen by our algorithm are the same as those of the OPT algorithm.

### 5.7. Algorithm Comparison

In Table 4, we compare the advantages of the four algorithms in terms of time complexity, global optimal solution and applicable scale.

## 6. Conclusions

In this paper, we considered a UAV-assisted shooting mission scenario. In order to improve the utility of shooting targets, we optimized UAV trajectory planning, hovering time and shooting point selection. By proving the submodularity of the utility function, we transformed the initial problem into a problem of maximizing the submodularity function under path constraints. We proposed an approximate algorithm to maximize shooting utility through two-level optimization, and the approximate ratio of the algorithm is proved to be $\left(1-1/e\right)/2$. Simulation results showed that the performance of our algorithm is closest to the optimal algorithm, and compared with the MSU and RAN algorithms, our proposed algorithm improves the overall shooting utility and time utilization efficiency. In this paper, we only consider the scenario where a single UAV completes the task, but multi-UAV cooperation can achieve more efficient shooting tasks. In the future work, we will consider the communication between UAVs and dynamically realize the task scheduling and trajectory planning of distributed UAVs by using deep reinforcement learning.

## Figures and Tables

**Table 1:** Notations.

Notation	Definition
O	Ground target set
C	Cluster head set
L	UAV trajectory set
*m*	Number of ground targets
*n*	Number of cluster heads
$d(c_{i},c_{i+1})$	Distance between cluster head *i* and *i* + 1
$E_{m}$	Motion energy cost of UAV
$E_{h}$	Hovering energy cost of UAV
$E_{max}$	Energy capacity of UAV
$v_{a}$	Velocity of UAV
*R*	Shooting radius of UAV
T	Time allocation strategy
K	Selected cluster head set
$Q(K,o_{j})$	Shooting utility for single target $o_{j}$
Q(K,T)	Overall Shooting utility

**Table 2:** Simulation Parameters.

Symbol	Definition	Value
*m*	Mass of UAV	2.07 kg
$E_{max}$	Energy capacity of UAV	4$\times 10^{4}$ J
*h*	Altitude of UAV	30 m
$v_{a}$	Velocity of UAV	20 m/s
$r_{u}$	Rotor radius	0.127 m
$n_{u}$	Number of rotors	4
ρ	Air density	1.225 kg/m${}^{3}$
*R*	Shooting radius of UAV	16 m

**Table 3:** Approximation of Shooting Utility Between UCR and OPT Algorithms Under Different Cluster Radius Parameters.

Radius of clusters (m)	10	11	12	13	14	15	16
Approximation of shooting utility	94.76%	94.91%	95.05%	94.69%	95.13%	95.19%	95.58%

**Table 4:** Algorithm Comparison.

Algorithm	Time Complexity	Global Optimal Solution	Applicable Scale
OPT	High	High	Small
UCR	Comparatively low	Comparatively high	Large
MSU	Low	Comparatively low	Large
RAN	Low	Low	Large

## Data Availability

Not applicable.

## Appendix A

According to the definition of the shooting utility function, shooting utility increases with an increase in the number of cluster heads. Thus, for any A⊆B⊆K, $G_{A}\left(o_{j}\right)\leq G_{B}\left(o_{j}\right)$. Due to the fact that the total shooting utility is superimposed, we obtain the following relationship:

(A1) $$G\left(A\right)=\sum_{j=1}^{m}G_{A}\left(o_{j}\right)\leq \sum_{j=1}^{m}G_{B}\left(o_{j}\right)=G\left(B\right),$$

which implies that G(K) is monotone.

From the definition of the substitution function, $G\left(K\right)=Q\left(K,T^{\prime }\right)\geq 0$, thus it is nonnegative.

For submodularity, we prove it by proving the following inequation $G\left(A+c_{i}\right)-G\left(A\right)\geq G\left(B+c_{i}\right)-G\left(B\right)$. It is equivalent to proving that for each target $o_{j}$, $G_{A+c_{i}}\left(o_{j}\right)-G_{A}\left(o_{j}\right)\geq G_{B+c_{i}}\left(o_{j}\right)-G_{B}\left(o_{j}\right)$. We use $G_{A+c_{i}}$ for $G(A\cup \left\{c_{i}\right\})$ to make the expression more intuitive.

It can be seen from the superposition of the shooting utility, $q_{A}\left(o_{j}\right)\leq q_{B}\left(o_{j}\right)$, then we can divide the proof problem into the following three cases.

**Case 1.** $q_{A}\left(o_{j}\right)\leq q_{B}\left(o_{j}\right)\leq Q_{j}^{m}$: In this case,

(A2) $$\begin{array}{cc} G_{A+c_{i}}\left(o_{j}\right)-G_{A}\left(o_{j}\right) & =\min\left\{q_{A+c_{i}}\left(o_{j}\right),Q_{j}^{m}\right\}-\min\left\{q_{A}\left(o_{j}\right),Q_{j}^{m}\right\}\\ \; & =\min\left\{q\left(o_{j},c_{i}\right),Q_{j}^{m}-q_{A}\left(o_{j}\right)\right\} \end{array}$$

and

(A3) $$\begin{array}{cc} G_{B+c_{i}}\left(o_{j}\right)-G_{B}\left(o_{j}\right) & =\min\left\{q_{B+c_{i}}\left(o_{j}\right),Q_{j}^{m}\right\}-\min\left\{q_{B}\left(o_{j}\right),Q_{j}^{m}\right\}\\ \; & =\min\left\{q\left(o_{j},c_{i}\right),Q_{j}^{m}-q_{B}\left(o_{j}\right)\right\}. \end{array}$$

Since $Q_{j}^{m}-q_{B}\left(o_{j}\right)\leq Q_{j}^{m}-q_{A}\left(o_{j}\right)$, we can consider the following three cases to prove the inequality. (a). If $q\left(o_{j},c_{i}\right)\leq Q_{j}^{m}-q_{B}\left(o_{j}\right)\leq Q_{j}^{m}-q_{A}\left(o_{j}\right)$, we have

(A4) $$G_{A+c_{i}}\left(o_{j}\right)-G_{A}\left(o_{j}\right)=q\left(o_{j},c_{i}\right)=G_{B+c_{i}}\left(o_{j}\right)-G_{B}\left(o_{j}\right).$$

(b). If $Q_{j}^{m}-q_{B}\left(o_{j}\right)<q\left(o_{j},c_{i}\right)<Q_{j}^{m}-q_{A}\left(o_{j}\right)$, we have

(A5) $$G_{A+c_{i}}\left(o_{j}\right)-G_{A}\left(o_{j}\right)=q\left(o_{j},c_{i}\right)>Q_{j}^{m}-q_{B}\left(o_{j}\right)=G_{B+c_{i}}\left(o_{j}\right)-G_{B}\left(o_{j}\right).$$

(c). If $Q_{j}^{m}-q_{B}\left(o_{j}\right)\leq Q_{j}^{m}-q_{A}\left(o_{j}\right)\leq q\left(o_{j},c_{i}\right)$, we have

(A6) $$G_{A+c_{i}}\left(o_{j}\right)-G_{A}\left(o_{j}\right)=Q_{j}^{m}-q_{A}\left(o_{j}\right)\geq Q_{j}^{m}-q_{B}\left(o_{j}\right)=G_{B+c_{i}}\left(o_{j}\right)-G_{B}\left(o_{j}\right).$$

**Case 2.** $q_{A}\left(o_{j}\right)\leq Q_{j}^{m}\leq q_{B}\left(o_{j}\right)$: In this case,

(A7) $$\begin{array}{cc} G_{A+c_{i}}\left(o_{j}\right)-G_{A}\left(o_{j}\right) & =\min\left\{q_{A+c_{i}}\left(o_{j}\right),Q_{j}^{m}\right\}-\min\left\{q_{A}\left(o_{j}\right),Q_{j}^{m}\right\}\\ \; & =\min\left\{q_{A+c_{i}}\left(o_{j}\right),Q_{j}^{m}\right\}-q_{A}\left(o_{j}\right)\\ \; & =\min\left\{q\left(o_{j},c_{i}\right),Q_{j}^{m}-q_{A}\left(o_{j}\right)\right\}\geq 0, \end{array}$$

(A8) $$\begin{array}{cc} G_{B+c_{i}}\left(o_{j}\right)-G_{B}\left(o_{j}\right) & =\min\left\{q_{B+c_{i}}\left(o_{j}\right),Q_{j}^{m}\right\}-\min\left\{q_{B}\left(o_{j}\right),Q_{j}^{m}\right\}\\ \; & =Q_{j}^{m}-Q_{j}^{m}=0. \end{array}$$

**Case 3.** $Q_{j}^{m}\leq q_{A}\left(o_{j}\right)\leq q_{B}\left(o_{j}\right)$: In this case,

(A9) $$\begin{array}{cc} G_{A+c_{i}}\left(o_{j}\right)-G_{A}\left(o_{j}\right) & =\min\left\{q_{A+c_{i}}\left(o_{j}\right),Q_{j}^{m}\right\}-\min\left\{q_{A}\left(o_{j}\right),Q_{j}^{m}\right\}\\ \; & =Q_{j}^{m}-Q_{j}^{m}=0, \end{array}$$

(A10) $$\begin{array}{cc} G_{B+c_{i}}\left(o_{j}\right)-G_{B}\left(o_{j}\right) & =\min\left\{q_{B+c_{i}}\left(o_{j}\right),Q_{j}^{m}\right\}-\min\left\{q_{B}\left(o_{j}\right),Q_{j}^{m}\right\}\\ \; & =Q_{j}^{m}-Q_{j}^{m}=0. \end{array}$$

For the above three cases, $G_{A+c_{i}}\left(o_{j}\right)-G_{A}\left(o_{j}\right)\geq G_{B+c_{i}}\left(o_{j}\right)-G_{B}\left(o_{j}\right)$ is satisfied, thus we prove that G(K) is a submodular function.
